# Lead editorial: The need for greater perspective and innovation in epidemiology

**DOI:** 10.1186/1742-5573-1-1

**Published:** 2004-09-03

**Authors:** Carl V Phillips, Karen J Goodman, Charles Poole, the editors of Epidemiologic Perspectives & Innovations

**Affiliations:** 1 University of Texas Health Science Center Houston 1200 Herman Pressler Dr., Houston, Texas 77030 USA; 2 Center for Philosophy, Health, and Policy Sciences, Inc., Houston, USA; 3 Department of Epidemiology, University of North Carolina, Chapel Hill, North Carolina, USA

## Abstract

This editorial introduces the new online, open-access, peer-reviewed journal, *Epidemiologic Perspectives & Innovations*. Epidemiology (which we define broadly, to include clinical research and various approaches to studying the health of populations) is a critically important field in informing decisions about the health of individuals and populations. But the desire for new information means that the health science literature is overwhelmingly devoted to reporting new findings, leaving little opportunity to improve the quality of the science. By creating a journal dedicated to all topics of and about epidemiology, except standard research reports, we hope to encourage authors to write more on the neglected aspects of the field. The journal will publish articles that analyze policy implications of health research, present new research methods and better communicate existing methods, reassess previous results and dogma, and provide other innovations in and perspectives on the field. Online publishing will permit articles of whatever length is required for the work, speed the time to publication and allow free access to the full content.

## 

Epidemiology is a critically important field in informing decisions about the health of individuals and populations. It is also a young field, with the potential for seeing fundamental improvements in the conduct of the science every year. But the desire for new information means that the health science literature is overwhelmingly devoted to reporting new findings, leaving little opportunity to improve the quality of the science. *Epidemiologic Perspectives & Innovations *(*EP&I*) was created to provide a forum for efforts to improve the quality of health science research and its applications.

Successful enterprises know they must devote a substantial portion of their resources – at least a few percent and often ten percent or more – to assessing whether the rest of their resources are being optimally directed. Such efforts include research and development, which improves the quality of products, and outcomes research, which assesses the impact of those products. In an applied science like epidemiology, these efforts should be devoted to designing new methods for conducting studies and interpreting results, translation of research into effective policy recommendations, critical review of past findings and current practice, and improvement of teaching the next generation of scientists.

Given the resources expended by the health science research enterprise, epidemiology (which we define broadly, including both population health and clinical research, and covering biological, behavioral, and economic dimensions) is characterized by remarkably little innovation, let alone critical review of existing dogma. A well-educated epidemiologist transported forward in time from 1980 would probably be able to read (and participate in) most current research and would find few surprises other than a few specific study results. Yet a substantial portion of all the epidemiology ever conducted has been carried out since 1980 – probably more than half, even including in the count all medical literature back to the dawn of literacy. The outputs of the science have increased to a torrent; research to improve the quality of the science is a trickle.

It can hardly be argued that this slow innovation and lack of perspective is because current approaches offer little room for improvement. Current discussions of advanced statistical methods, the nature of random error, sensitivity analysis and uncertainty quantification, and proper interpretation of results, to name just a few, show that most current epidemiologic research uses methodology in need of improvement. Granting that many problems would be eliminated if health researchers – who often have minimal training in epidemiology – just followed the dictates of a good basic epidemiology textbook, there are still major problems that lack simple solutions. It is troubling that we plow ahead with billions of dollars worth of research every year while making minimal effort to answer fundamental questions about what that research is really telling us. Epidemiology is far too important to our society to be treated as an exercise in uncritically following existing formulae.

The limitations of the field become even more apparent after the research is conducted. Results are cast out into the world as if they speak for themselves, forcing policy makers, clinicians, and interested lay people to interpret them, despite their lack of expertise in the topic area and analytic methods, and lack of necessary context.

### A missing marketplace for ideas

These problems leave plenty of blame to go around, but a fair amount of it rests with the lack of opportunity to publish scholarly analytic work aimed at solving the problems. While some health researchers might be guilty of not giving the field's limitations a second thought, most probably can envision contributions they would like to make to improve it. Every conclusions section containing a single paragraph of policy discussion suggests that researchers would like to contribute to policy analysis, but have exhausted their paltry word limit. Every dissertation that reflects upon and challenges standard methods shows fresh analytic thinking, but the innovations might be read by just the half dozen people who view the actual dissertation, since the resulting publications will likely be limited to a brief recounting of the methods and results (narrowly defined). Every time a professor explains to her students that something they learned in a previous class or from reading the literature is wrong, there is a lesson that should be getting out to everyone in the field, not just the ten students in that room.

These scenarios call for full-length, analytically complete presentations (see endnote 1). Such analysis cannot be grafted onto a paper primarily focused on presenting numerical results from a study, given the severe length limitations in print journals in the health sciences. Indeed, such analyses must usually be longer than typical word limits allow, even without the study results or applications that are needed to illustrate them or provide background. A researcher who writes such a paper has a very difficult time getting it published. Moreover, it is easy to anticipate that difficulty and never even try to write the paper. The founding editors of this journal were inspired by their own experiences with these difficulties.

There are journals that cover some of these areas, but to a remarkably limited extent. "Health policy" (and more so, "health economics") journals focus on the financial side of health care, rather than more general health policy or economic issues. Statistics journals, and even the research methodology slots available in the health research journals, favor mathematically complicated advances over more practical advice. With the exception of occasional relevant entries in medical journal education series, articles devoted to improving the teaching or understanding of epidemiologic research have no clear venue. Even with some niches for certain methods, policy, overview, or perspectives articles, it is difficult to be enthusiastic about writing in these areas with no clear idea of where the results are likely to be published.

*Epidemiologic Perspectives & Innovations *(*EP&I*) was created to provide this forum, taking advantage of the greater visibility and article length offered by open-access online publishing.

## Article topics

The following are areas of inquiry *EP&I *will publish. (An accompanying editorial [1] presents a more specific "wish list" of some of the particular analyses the editors would like to see.)

Policy: Policy recommendations do not flow simply and directly from health research results, and educated recommendations demand more than a few sentences of analysis at the end of a research report. A good policy recommendation requires high-quality analysis of a nature and quantity that does not fit in standard health research journals. At the same time, health researchers cannot leave policy analysis of their results for other people to do and publish in policy journals because there are very few such people or journals. If health researchers do not take the lead on policy analyses based on their research, the analyses will likely never be done. *EP&I *fills the gap by providing a forum for policy analysis in the context of health research. Policy analysis articles can be free-standing or specifically based on research reports published elsewhere. Submissions in this area should be analytic (addressing policy/decision analysis, economics, ethics, or other areas of analytic inquiry), rather than commentary.

Methodologic Innovation and Communication: *EP&I *welcomes submissions in all areas of epidemiologic research methodology, from study design to data analysis and reporting, including new tools, simple but important observations, and widely understandable applications of existing tools. The strength of our editorial board in this area means that submissions will be reviewed by experts who understand and appreciate new methods. Unlike most other journals publishing methods articles, *EP&I *welcomes submissions that are not necessarily at the technological cutting edge (though such submissions are also encouraged), but that contain lessons that are not widely known. We will spare authors the all-too-common experience of being told "everyone already knows that" when they submit a paper that calls for the use of methods or practices that are widely overlooked. Research articles are needed to translate methodological findings that are "known" (in the sense of having been discovered and understood by methods specialists), to make them known (in the sense of being understood and usable by most researchers in the field).

Ethics, Philosophy, and Critical Analysis of the Field: In most of the health research literature, any discussion of philosophical points or assessments of the quality of research is labeled "commentary" and restricted to the opinions of a few luminaries. But carefully reasoned ethical analysis, epistemology, analysis of quality, and the like are not mere commentary, and often come from junior researchers or outsiders. Our accompanying "wish list" editorial provides some examples of these types of analysis.

Re-analyses: The deluge of research results in health science means that few study results are ever carefully re-analyzed, even when their implications are quite important. When such re-analyses do occur, they are often limited to letters or perfunctory assessments in systematic reviews. *EP&I *offers a forum for publishing full-length re-analyses (which might use different analytic approaches, start with different premises, or report different results) of important previous research.

Teaching Methods and Innovations: Many fields have a dedicated teaching section in one or more journals. *EP&I *will include articles that provide teaching tools, innovations, and methods. Online publishing allows authors to include computer code, spreadsheets, datasets, and other tools that will allow readers to make use of the teaching tools. Teaching articles will be peer reviewed by experienced teachers and at least one current student at the appropriate level to judge the material. The ideal teaching articles will present an approach or method, the specific tools necessary for a reader to implement it, and a report of the authors' experience in using the material. Review will be based primarily on the apparent usefulness of the presented approach.

Multidisciplinary Research: This category is somewhat redundant, given that many of the aforementioned article types necessarily draw upon knowledge from multiple disciplines. But it is worth mentioning specifically because it is often difficult to publish work that is based in multiple fields of inquiry and thus does not fit easily into any one of them, or that is squarely in another discipline but is intended for an audience of health scientists. *EP&I *encourages such submissions and will review them based on their analytic merits in the fields in which they are based and their potential usefulness to health researchers.

More generally, *EP&I *is a home for all articles of and about epidemiology, with the exception of standard research reports. (Reporting research results as part of one of the above article types is, of course, welcome.) This includes many types of papers that are not themselves epidemiologic analysis, but inform epidemiology or are about epidemiology. We suspect that many papers of the above types exist on paper or in researchers' heads, but have previously been difficult to get published. Many more will be written when they are appreciated as analytic work that is central to the field.

## Flexible format

To provide maximum flexibility for these kinds of articles, we worked with BioMed Central to create the "Analytic Perspectives" article type. We expect most submissions to *EP&I *to be this article type, which allows authors to create a structure that fits their analysis (as opposed to methods-results-discussion, which generally would not fit) and is labeled to emphasize that the article is analytic (as opposed to commentary).

We are also taking the unusual (for a health science journal) step of encouraging the use of endnotes. We believe that the lack of substantive endnotes or footnotes – to provide important asides, definitions, or clarifications, to note exceptions to general rules, or provide other elaboration – is a major detriment to the content of health research papers, making it difficult to present certain analytic points. For example, an interesting statistical claim or policy observation that is almost always true can either be made without further elaboration (in which case the exceptions make the claim incorrect), can include a paragraph of caveats (which is awkward and distracting), or left out (see endnote 2). Often the latter is the author's choice, which impoverishes the literature. An endnote could solve the problem. In other cases, endnotes could include short derivations of calculations that will be obvious to some readers and uninteresting to others, but may be of interest to some. Anyone familiar with the social science literature or many other fields will understand the beneficial uses to which such notes can be put (see endnote 3). Authors should consult the instructions for submissions for mechanical details of endnote use.

## Target audience and authors

We hope that many readers will read *EP&I *from virtual cover to virtual cover. Most readers of most health science journals scan the table of contents and read the one or two articles that report results in their subspecialty, or never even see a table of contents, but merely a list of search hits on PubMed. Most articles in *EP&I *should be of some interest to researchers who are serious about understanding analytic health research and its implications.

We welcome submissions from researchers with all levels of experience in the field, and from experts in other fields writing for health researchers. Great innovations and critical analysis often come from senior scholars in a field, but they also often come from graduate students, outsiders, and others who are not heavily invested in the status quo of a field of inquiry.

## Conclusions

In 1995, *Science *published the controversial article, "Epidemiology Faces its Limits" [2], which suggested that the field had already gathered all the low-hanging fruit and was not able to do much more. The premise implied by the title was dead wrong and still is: epidemiologic research (whether defined broadly or more narrowly) is no where near the limits of its technology or potential contribution to our knowledge. But nearly a decade later, the criticisms that rang true when that article was published still ring true; the progress toward "breakthroughs in the methodological tools of epidemiology" called for in that article has been limited. *EP&I *hopes to encourage the pursuit of breakthroughs (or, better still, a slow and steady flow of new innovations and perspectives) by providing a ready home for publishing a broad collection of such material.

## Endnotes

1. "Analytic" should not be confused with quantitative calculations and results, which seldom contain much actual analysis. Analysis can be thought of as the intellectual process of systematic inquiry aimed at understanding, explaining, or characterizing phenomena or concepts.

2. For example, authors might want to point out that nondifferential misclassification error that they suspect exists most likely biased a result toward the null. However, an unqualified statement to this effect will likely generate criticism that such error is not always toward the null and that believing so is a sure sign of methodological naivete. An endnote in which the authors point out that there are exceptions, but the bias is still usually toward the null, would allow them to make their point without a long awkward caveat breaking up the main text.

3. The literature in most of these fields uses footnotes, conveniently located at the bottom of the page. Online publishing leaves us without a bottom of the page, but allows for convenient opening of multiple windows, offering the opportunity to have the endnotes open in a separate browser window. Readers of printed PDF versions will, alas, have to flip to the end.
